# Strategies for the prevention of autoimmune Type 1 diabetes

**DOI:** 10.1111/j.1464-5491.2011.03400.x

**Published:** 2011-10

**Authors:** J A Todd, M Knip, C Mathieu

**Affiliations:** * University of CambridgeUK; † Children's Hospital, University of HelsinkiHelsinki, Finland; ‡ Katholieke Universiteit LeuvenBelgium

## Abstract

European experts on autoimmune Type 1 diabetes met for 2 days in October 2010 in Cambridge, to review the state-of-the-art and to discuss strategies for prevention of Type 1 diabetes (http://www-gene.cimr.cam.ac.uk/todd/sub_pages/T1D_prevention_Cambridge_workshop_20_21Oct2010.pdf). Meeting sessions examined the epidemiology of Type 1 diabetes; possible underlying causes of the continuing and rapid increase in Type 1 diabetes incidence at younger ages; and lessons learned from previous prevention trials. Consensus recommendations from the meeting were:

1. Resources such as national diabetes registries and natural history studies play an essential role in developing and refining assays to be used in screening for risk factors for Type 1 diabetes.
2. It is crucial to dissect out the earliest physiological events after birth, which are controlled by the susceptibility genes now identified in Type 1 diabetes, and the environmental factors that might affect these phenotypes, in order to bring forward a mechanistic approach to designing future prevention trials.
3. Current interventions at later stages of disease, such as in newly diagnosed Type 1 diabetes, have relied mainly on non-antigen-specific mechanisms. For primary prevention—preventing the onset of autoimmunity—interventions must be based on knowledge of the actual disease process such that: participants in a trial would be stratified according the disease-associated molecular phenotypes; the autoantigen(s) and immune responses to them; and the manipulation of the environment, as early as possible in life. Combinations of interventions should be considered as they may allow targeting different components of disease, thus lowering side effects while increasing efficacy.

Type 1 diabetes occurs when the body's immune system turns against itself so that, in a very specific and targeted way, it destroys the pancreatic islet β-cells, the only cells in the body that produce the vital hormone insulin. This autoimmune destruction is irreversible and the disease incurable. If new pancreas or islets are transplanted they too are destroyed, unless heavy immunosuppression is applied. Recent genetics and mechanistic studies in humans are indicating major pathways that play roles in early disease development, especially in the interleukin-2 (IL-2) and type 1 interferon systems, probably in concert with the immune response to preproinsulin [1,2].

Longitudinal studies of children born with high genetic risk of Type 1 diabetes showed that circulating autoantibodies to islet antigens can be seen as early as 6 months. A large proportion of childhood cases has seroconverted by age 3 years, and almost all cases by age 7 years, even although median age-at-diagnosis is approximately 12 years [3,4]. Children who will go on to develop Type 1 diabetes in the future must be born with the first precursors of the disease. A child aged under age 5 years with a family history of Type 1 diabetes, carrying the highest risk human leukocyte antigen (HLA) class II genotypes and persistently positive for two or more autoantibody types, has a more than 90% chance of being diagnosed with the disease [5]. By making the connection between gene variant and phenotype, genetics provides a way to identify pathways leading to Type 1 diabetes, including the earliest physiological events after birth, and the earliest events in the disease process, including clues about what may be environmental factors or accelerators of disease [4,6].

National diabetes registries, especially those with available biosamples, provide Europe with important population-based research resources, for studying disease epidemiology, or for testing hypotheses about disease initiation and progression. Registry data where available provide evidence that the incidence of Type 1 diabetes in children under age 15 years has increased. However, there is a shortage of data on Type 1 diabetes over age 15, although at least half of new cases occur after age 15. Data from registries should be accessible to all bona fide researchers, provided that necessary confidentiality protections are in place. Common standards regarding data fields would add value and also aid data access.

The appearance of autoantibodies, the first of which chronologically are usually against insulin itself, is a secondary or downstream event in the autoimmune process. Identification and validation of biomarkers that precede autoantibody appearance are urgently required. Incentives to provide access to these collections should be implemented [7]. Useful information to provide possible investigators with includes limitations of the informed consent, types and amounts of material available and storage conditions. Efforts should be made to extend collections of samples of at-risk individuals and persons with newly diagnosed diabetes. Funding opportunities should be implemented to encourage investigators to develop assays that could be used retrospectively, to employ new technologies and to use smaller assay volumes for studying markers.

Despite the limited success of previous prevention trials, they have provided valuable lessons, combined with correctly interpreted data from the animal models. Predictions of the natural history of disease based on combined genetic (HLA) and serological markers (autoantibodies) are accurate. C-peptide is a surrogate marker for loss of β-cell function and progression to diabetes; and the oral glucose tolerance test is a sensitive screening tool for detecting individuals with diabetes before fasting blood sugar or HbA_1c_ levels become abnormal. Screening for risk of diabetes can avoid suffering in children, as diabetic ketoacidosis prevalence was 4% in children enrolled in prevention trials compared with 15–20% in new-onset children in the general population [8]. Interventions at later stages of disease, such as in newly diagnosed Type 1 diabetes, have used mainly non-antigen-specific drugs, with the promising studies having used doses at the edge of safety. Successfully preventing the onset of autoimmunity—true primary prevention— must include antigen-specific tolerance approaches [2]. It is important to begin now to accurately establish doses, timing and best route of administration, whilst analysing mechanistic effects.

## Workshop participants

Participants of the *Expert Workshop on the Rising Incidence of T1D in Europe*, organized by the UK Science and Innovation Network in collaboration with JDRF, 20–21 October 2010, Cambridge, UK:

Dr Peter Achenbach, Diabetes Research Institute, Munich, Germany

Professor Polly Bingley, School of Clinical Sciences, University of Bristol, UK

Dr Ezio Bonifacio, DFG Centre for Regenerative Therapies, Dresden, Germany

Rachel Connor, Head of Research Communication, JDRF, UK

Dr Achmed Delli, Department of Clinical Sciences/Diabetes and Celiac Disease, Lund University

Dr Karen Finney, Programme Manager, Nutrition and Metabolic Medicine, Medical Research Council, UK

Dr Iain Frame, Diabetes UK, London, UK

Dr Staffan Fraysse, Medical and Public Health Research, DG Research, European Commission

Professor Frans Gorus, Diabetes Research Centre, Brussels Free University, Belgium

Graham Hughes, National Contact Point FP7

Dr Richard Insel, Chief Scientific Officer, JDRF, USA

Professor Desmond Johnston, Department of Medicine, Imperial College London, UK

Professor Mikael Knip, Professor of Paediatrics, University of Helsinki, Finland

Professor Chantal Mathieu, Director Endocrinology Clinic, University Hospital Leuven, Belgium

Dr Clare McVicker, Molecular and Physical Sciences, Wellcome Trust, UK

Dr Concepcion Nierras, Director of Partnerships and Consortia, JDRF, USA

Dr Chris Patterson, Queen's University Belfast, UK

Professor Mark Peakman, King's College London, UK

Professor Flemming Pociot, Genome Biology Team, Hagedorn Research Institute, Denmark

Professor Paolo Pozzilli, Centre for Diabetes, London School of Medicine, UK

Professor Dr Bart Roep, Diabetes and Immunology, Leiden University Medical Centre, the Netherlands

Dr Kjersti Ronningen, Norwegian Institute of Public Health, Oslo, Norway

Professor Dr Eugen Schoenle, Pediatric Endocrinology and Diabetology, Zurich University Children's Hospital, Switzerland

Professor Olli Simell, Department of Paediatrics, University of Turku, Finland

Dr Teodora Staeva, Director of Immunology, JDRF, USA

Professor John Todd, JDRF/Wellcome Trust Diabetes and Inflammation Laboratory, Cambridge University, UK

Dr Outi Vaarala, Department of Molecular Medicine, National Institute for Health and Welfare, Helsinki, Finland

Professor Nick Wareham, MRC Epidemiology Unit, Institute of Metabolic Science, Addenbrooke's Hospital, Cambridge, UK

Professor Linda Wicker, JDRF/Wellcome Trust Diabetes and Inflammation Laboratory, Cambridge University, UK

Professor Dr. Anette G. Ziegler, University of Technology and Helmholtz Centre, Munich, Germany

## References

[1] Todd JA (2010). Etiology of type 1 diabetes. Immunity.

[2] Skowera A, Ellis RJ, Varela-Calvino R, Arif S, Huang GC, Van-Krinks C (2008). CTLs are targeted to kill beta cells in patients with type 1 diabetes through recognition of a glucose-regulated preproinsulin epitope. J Clin Invest.

[3] Siljander HT, Simell S, Hekkala A, Lähde J, Simell T, Vähäsalo P (2009). Predictive characteristics of diabetes-associated autoantibodies among children with HLA-conferred disease susceptibility in the general population. Diabetes.

[4] Winkler C, Lauber C, Adler K, Grallert H, Illig T, Ziegler AG, Bonifacio E (2011). An interferon-induced helicase (IFIH1) gene polymorphism associates with different rates of progression from autoimmunity to type 1 diabetes. Diabetes.

[5] Bingley PJ, Gale EA (2006). Progression to type 1 diabetes in islet cell antibody-positive relatives in the European Nicotinamide Diabetes Intervention Trial: the role of additional immune, genetic and metabolic markers of risk. Diabetologia.

[6] Knip M, Veijola R, Virtanen SM, Hyoty H, Vaarala O, Akerblom HK (2005). Environmental triggers and determinants of type 1 diabetes. Diabetes.

[7] Hagopian WA, Erlich H, Lernmark A, Rewers M, Ziegler AG, Simell O (2011). The Environmental Determinants of Diabetes in the Young (TEDDY): genetic criteria and international diabetes risk screening of 421 000 infants. Pediatr Diabetes.

[8] Nanto-Salonen K, Kupila A, Simell S, Siljander H, Salonsaari T, Hekkala A (2008). Nasal insulin to prevent type 1 diabetes in children with HLA genotypes and autoantibodies conferring increased risk of disease: a double-blind, randomised controlled trial. Lancet.

